# Clinicopathological relevance of stem cell marker growth and differentiation factor 3 in esophageal squamous cell carcinoma

**DOI:** 10.37349/etat.2023.00130

**Published:** 2023-04-24

**Authors:** Sara Tahbazzadeh Moghaddam, Mohammad Mahdi Forghanifard

**Affiliations:** 1 Division of Biotechnology, Faculty of Veterinary Medicine, Ferdowsi University of Mashhad, Mashhad 9177948974, Iran; 2 Department of Biology, Damghan Branch, Islamic Azad University, Damghan 3671639998, Iran; Huazhong University of Science and Technology, China

**Keywords:** Cancer stem cell marker, esophageal squamous cell carcinoma, growth and differentiation factor 3, real-time polymerase chain reaction, expressional analysis

## Abstract

Esophageal squamous cell carcinoma (ESCC) is the second leading cause of cancer-related deaths in Iran, often diagnosed in advanced stages with a poor prognosis. Growth and differentiation factor 3 (*GDF3*) is a member of the transforming growth factor-beta (TGF-β) superfamily. It acts as an inhibitor of bone morphogenetic proteins (BMPs) signaling pathway associated with pluripotent embryonic and cancer stem cells (CSCs) characteristics. Since its expression in ESCC has not yet been evaluated, the clinicopathological relevance of *GDF3* expression was elucidated in ESCC patients. Expression of *GDF3* in tumor tissues from 40 ESCC patients was compared to the related margin normal tissues by relatively comparative real-time polymerase chain reaction (PCR). Glyceraldehydes 3-phosphate dehydrogenase (*GAPDH*) was used as the endogenous control. Likewise, the function of *GDF3* in the differentiation and development of embryonic stem cells (ESCs) was also reviewed. *GDF3* was significantly overexpressed in 17.5% of tumors and a significant correlation between *GDF3* expression and the depth of tumor invasion was observed (*P* = 0.032). The results suggest that *GDF3* expression is likely to have substantial roles in the progression and invasiveness behavior of ESCC. Having considered the importance of CSC markers identification and their exploitation in targeted cancer therapy, *GDF3* may be introduced as a promising therapeutic target to inhibit the invasion of tumor cells in ESCC.

## Introduction

Esophageal carcinoma (EC) is the eighth most common cancer and the sixth most prevalent cause of cancer-related deaths, worldwide [1]. Esophageal squamous cell carcinoma (ESCC) is the most predominant type of EC globally [2] and the second leading cause of cancer-related mortality in northeastern Iran, a high-risk area located on the “Central Asian Esophageal Cancer Belt” that extends from China to northeastern Iran. Most ESCC cases are presented at advanced stages, due to delayed diagnosis and poor prognosis [3, 4].

Cancer stem cells (CSCs) have been defined as cells within a tumor that possess the exclusive ability to self-renew and generate the heterogeneous lineages of cancer cells that contain the tumor [5]. The transformation of a normal stem cell into a CSC is due to the accumulation of genetic modifications and epigenetic alterations [6]. CSCs are responsible for tumor progression, resistance, and recurrence that lead to the failure of traditional cancer therapies [7]. Transforming growth factor-beta (TGF-β) is a large family of secreted proteins that are involved in a wide range of cellular processes such as proliferation, differentiation, migration, and apoptosis. Members of this family have fundamental roles in embryonal development, tissue repair, and immune system modulation [8]. TGF-β family consists of two main classes: the TGF-β/activin/ nodal and the bone morphogenetic protein (BMP)/growth and differentiation factor (GDF).

GDF3 is a ligand of the BMP/GDF branch associated with pluripotent embryonic stem cells (ESCs) which was firstly cloned from a mouse embryonic day 6.5 (e6.5) complementary DNA (cDNA) embryonic library by homology to Xenopus Vg-1, another TGF-β family member. It has been shown that GDF3 is a bi-functional ligand regulating the balance between the two modes of TGF-β signaling. It may act as a BMP inhibitor at low doses if present as a prepro form or mature heterodimers with BMPs, while its homodimer form at high doses may act as a nodal-type agonist or activating ligand [9, 10]. *GDF3* is demonstrated as an important player in cancer biology. It is reported that *GDF3* regulates the expression of genes involved in differentiation, but does not influence the proliferative capacity of undifferentiated CSC. Furthermore, it is confirmed that *GDF3* protects the CSC from apoptosis induced by retinoic acid exposure [11]. *GDF3* regulates both of the two major characteristics of ESCs: the ability to maintain an undifferentiated state and the capacity of cell differentiation into the all-cell types of an organism [12].

*GDF3* is expressed in several malignancies such as melanoma [13], teratocarcinoma [14], testicular germ cell tumors (TGCTs) [15], seminoma, and breast carcinoma [16, 17]. Having considered the importance of CSC markers identification and their exploitation in targeted cancer therapy, since there is not any evidence reporting the expression of *GDF3* in ESCC, the expression of *GDF3* in the disease and its correlation with clinicopathological features of the patients were elucidated. Besides, the function of *GDF3* in the differentiation and development of ESCs to decipher its presumptive roles in the progression and invasiveness of ESCC was reviewed.

## Tissue collection, RNA extraction, and relative comparative real-time- polymerase chain reaction

### Tissue samples

In this retrospective study, the fresh tumor and its corresponding normal margin of ESCC tissues were collected from 40 new case patients who underwent curative surgery at Omid Oncology Hospital of Mashhad University of Medical Sciences (MUMS), Iran, between 2018 and 2021. All patients did not receive any chemo- or radio-therapy before the surgery and gave their informed consent to be enrolled in the study. The ethics committee of MUMS approved the study. Samples were immediately transferred to RNA later solution (Qiagen, Hilden, Germany) and stored at –20°C until RNA extraction. Tumor specimens were microscopically examined by expert pathologists to ensure containing at least 70% tumor cells. Normal tissues were also confirmed histologically. Furthermore, histopathological features of tumor samples including tumor size, location, and grade of differentiation were defined, and the surgical stage was determined on the basis of the seventh edition of Union International Cancer Tumor-Node-Metastasis (TNM) classification guidelines [18].

### RNA extraction, cDNA synthesis, and quantitative real-time-polymerase chain reaction

RNA was extracted from fresh normal and tumor tissues of the patients using the RNeasy Mini kit (Qiagen, Hilden, Germany). The quantity and purity of the isolated RNA samples were evaluated using spectrophotometry and agarose gel electrophoresis. Reverse transcription (RT) of total RNA with oligo dT was carried out as described before [19]. Relative comparative real-time polymerase chain reaction (PCR) was performed by SYBR Green PCR Master Mix (Fermentas, Lithuania), containing Rhodamine X (ROX) as a reference dye in a Stratagene Mx3000P real-time thermocycler (Stratagene, La Jolla, CA), with primers presented in Table 1. The thermal profile included 10 min at 95°C followed by 40 cycles of 30 s at 95°C, 30 s at 59°C, and 30 s at 72°C. Glyceraldehydes 3-phosphate dehydrogenase (*GAPDH*) was applied as an endogenous control to normalize the data [20]. The PCR efficiency for *GAPDH* and *GDF3* genes was measured using related standard curves generated by serial dilution of cDNA. The reactions were accomplished in triplicate. Overexpression was considered as a more than 2-fold fluorescence intensity of messenger RNA (mRNA) expression in the tumor tissue in comparison with adjacent normal tissues and underexpression was less than a minus 2-fold intensity. The range in-between was defined as normal expression.

**Table 1.** Primer sequences used for quantitative real-time PCR

**Gene**	**Forward primer sequence**	**Reverse primer sequence**
*GDF3*	TGTCTGCCATCAAAGAAAGGGAAC	GGGACTGACCGCAACACAAAC
*GAPDH*	GGAAGGTGAAGGTCGGAGTCA	GTCATTGATGGCAACAATATCCACT

### Statistical analysis

Data were analyzed using the SPSS 19.9 statistical package (SPSS, Chicago, IL). The *χ*^2^ or Fisher exact test, independent-sample *t* test, and analysis of variance (ANOVA) were employed to explore the associations between *GDF3* expression levels and various clinicopathological factors. *P* < 0.05 was considered to be statistically significant.

## *GDF3* overexpression in ESCC and its correlation with depth of tumor invasion

### Study population

A total of 40 newly-diagnosed and histologically corroborated ESCC patients were enrolled in this study including 20 males (50%) and 20 females (50%). The mean [± standard deviation (SD)] age of the patients was 60.94 (± 11.50) years ranging between 30 and 80. The male-to-female ratio was 1:1. The size of tumor samples ranged from 1.50 cm to 8.00 cm with a mean (± SD) size of 3.99 (± 1.57) cm. Samples were resected from the middle (21/40, 52.5%) or lower (19/40, 47.5%) regions of the esophagus. Twenty-six of 40 tumor tissues (65%) were in stages I/II of tumor cell progression, while 14 of 40 (35%) showed advanced stages (III/IV). Most of the tumor tissues (31/40, 77.5%) showed invasion of the adventitia [tumor invasion level 3 (T3), 4]. In addition, 18 of 40 tumor samples (45%) were metastatic to the local lymph nodes. The clinicopathological characteristics of the patients are summarized in Table 2.

**Table 2.** Clinicopathological features of the ESCC patients and their correlation with *GDF3* gene expression

**Factor**	**Number (%)**	***GDF3***		***P* value**
		**Overexpression**	**Unchanged or underexpression**	
Tumor invasion				0.032^*^
T1, 2	9 (22.5)	1	8	-
T3, 4	31 (77.5)	6	25	-
Metastasis				0.964
No metastasis	22 (55)	3	19	-
Node metastasis	18 (45)	4	14	-
Stage				0.957
I/II	26 (65)	3	23	-
III/IV	14 (35)	4	10	-
Grade				0.450
WD	8 (20)	2	6	-
MD	28 (70)	4	24	-
PD	4 (10)	1	3	-
Tumor location				0.828
Middle	21 (52.5)	3	18	-
Lower	19 (47.5)	4	15	-
Sex				0.174
Male	20 (50%)	4	16	-
Female	20 (50%)	3	17	-

-: blank cell; ^*^significant correlation; WD: well differentiated; MD: moderately differentiated; PD: poorly differentiated

### Upregulation of *GDF3* in ESCC

**Figure 1.**
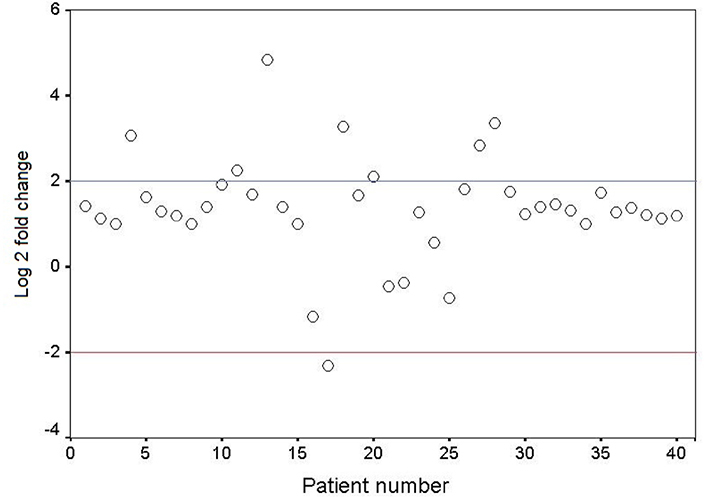
Scatter plot representative of descriptive analysis of relative gene expression distribution of *GDF3* in ESCC patients. The Y axis indicates the relative gene expression, and the X axis represents the patients. Relative mRNA expression of more than two-fold in tumor tissues is considered overexpression, less than minus two-fold as underexpression, and the range in-between is interpreted as normal. Log 2: logarithm 2

**Figure 2.**
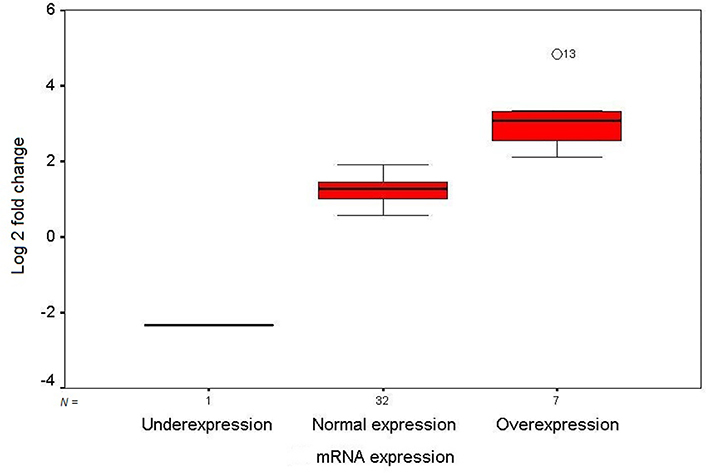
Box plot illustration of *GDF3* relative quantitative mRNA expression in ESCC patients. The Y axis indicates the fold change of relative mRNA expression, and the X axis shows patient groups. Box plots represent the lowest, lower quartile, median, upper quartile, and highest observations of fold changes in patients with underexpression, normal expression, and overexpression of *GDF3*. Patient number 13 showed an intense overexpression of the gene in tumor tissue (out layer 13; O13)

The mRNA expression level of *GDF3* in the fresh frozen neoplastic tissue was compared to the distant resected tumor-free esophageal epithelium by quantitative real-time RT-PCR. The expression pattern of the *GDF3* gene in the patients is depicted as a scatter plot in Figure 1. Overexpression of *GDF3* mRNA was observed in 7 of 40 tumor samples (17.5%). The minimum and maximum mRNA expression fold changes were −2.32 and 4.83, respectively [mean (± SD): 1.35 (± 1.23)]. The expression levels in patients with normal/underexpressed and overexpressed *GDF3* were compared together schematically in Figure 2.

### Association of *GDF3* expression with clinicopathological features

To assess the clinicopathological outcomes of *GDF3* expression in ESCC, the relevance of different clinicopathological variables with *GDF3* mRNA level was investigated (Table 2). The expression of *GDF3* mRNA was significantly correlated with the depth of tumor invasion (*P* < 0.05). Excluding for 1, all of the patients with *GDF3* overexpression had the invasion of tumor cells to the adventitia (6 of 7), which emphasizes the probable role of this factor in the invasiveness of the disease. Interestingly, 8 of 9 tumors without invasion to the adventitia (T1, 2) were not overexpressed for *GDF3* too. Although there was not any significant association between *GDF3* expression and other clinicopathological aspects such as tumor stage, grade of differentiation, and lymph node metastasis, 4 of 7 *GDF3* overexpressed tumors (57.1%) were in advanced stages (III/IV), MD, showing lymph node metastasis. Furthermore, 86.3% of patients (19 of 22) with no metastasis of tumor cells to the lymph nodes did not have *GDF3* overexpression.

## Discussion

ESCC is an extremely aggressive malignancy, with early lymphatic and hematogenous metastasis as well as a poor prognosis. The consequence of ESCC conventional treatments including surgery, radiotherapy, chemotherapy, or a combination of these procedures has not been improved noticeably [21]. Therefore, novel differential diagnostic markers and personalized treatment are required [22]. CSCs are hypothesized to cause the initiation, progression, and relapse of cancer. CSC markers may be utilized to identify CSCs in tumor mass and studying their role in tumorigenesis may help to improve therapeutic modalities [23]. Analysis of CSCs markers’ expressional pattern in ESCC and evaluating their molecular mechanism involved in tumorigenesis, makes it possible to develop targeted approaches for diagnosis and treatment of this malignancy.

In this study, the expression level of the *GDF3* in ESCC patients was evaluated and its overexpression was found in 17.5% of tumor tissues at the mRNA level in significant correlation with the depth of tumor cell invasion. Moreover, 85.7% of GDF3 overexpressed samples were invaded to the adventitia (T3, 4), emphasizing the remarkable role of GDF3 overexpression in tumor progression and invasion of ESCC.

GDF3 expression has been studied in different malignancies with contradictory reports. Nonetheless, the role of GDF3 in cancer biology remains poorly understood. It has been indicated that GDF3 augments the progression of B16 melanoma [13] and can enhance neuronal differentiation of PC12 cells [24], but inhibits the proliferation of breast carcinoma cell line MCF7. It has been shown that the GDF3 knockdown in human breast cancer cells can cause the propagation of colony formation and tumor progression, while its overexpression can promote apoptosis [17]. In support of this evidence, recently it is revealed that GDF3 expression was limited to 7.7% (9/117) of primary breast tumor samples and was correlated with the absence of axillary lymph node metastasis, suggesting a protective effect of GDF3 [25]. In addition, using NCCIT cells (a developmentally pluripotent permanent cell line derived from a mediastinal nonseminomatous germ cell tumors), as a system with CSC-like properties, Tykwinska et al. [11] reported the protective effect of GDF3 against retinoic acid-induced apoptosis in CSCs. It has been determined that in primary TGCTs GDF3 expression is low in seminomas, while its expression in non-seminomas is easily acquirable and appeared to be correlated with the embryonal carcinoma and yolk sac components in the tumors [15]. Furthermore, seminomas have elevated levels of GDF3 expression compared to the normal testis, as well as other stem cell markers such as Nanog homeobox (NANOG) and octamer-binding transcription factor 4 (OCT4), and in particular DAZL (a germ cell-specific marker) [16].

It has been clearly demonstrated that human stella-related (*STELLAR*), *NANOG*, and *GDF3* genes are expressed in pluripotent cells and mapped to chromosome 12p13, which is a hotspot for teratocarcinoma [14]. Indeed, this region is pointed to the stem cell genes as potential key oncogenes. These findings suggested that the 12p stem cell cluster and in particular *NANOG*, *STELLAR*, and *GDF3* may be commonly involved in the tumorigenesis process of different malignancies as well as constitute effective candidates and potential target genes for cancer therapy [26].

In ESCs, *GDF3* is obligatory for usual development *in vivo* and *in vitro*. It was substantiated that *GDF3* affects the expression of involved genes in different developmental processes and cell signaling pathways. Additionally, it strongly contributed to ectoderm/mesoderm development, neurogenesis, and negative angiogenesis regulation, representing the incorporation of the multi-differentiation potential of target cells [11]. During primary embryogenesis, *GDF3* is predominantly expressed in the inner cell mass of the blastocyst and plays role in mesoderm and deterministic endoderm organization in the pre-gastrulation stages of development. It has been shown also that *GDF3* is expressed during embryonic bone development as well as in the brain, thymus, spleen, bone marrow, and adipose tissues of adults [12]. Apart from the key characterization of *GDF3* as a stem cell marker, it is currently introduced as an adipogenic cytokine that is associated with the obesity process [24].

*GDF3* is exclusively expressed in the pluripotent and undifferentiated states, and conflictingly controls the differentiation of mouse and human ESCs. *In vitro* studies disclosed that *GDF3* has a species-dependent role in ESCs. Indeed, a high level of *GDF3* expression protects the stemness state in human ESCs, while its low level maintains the stemness state of mouse ESCs [12]. These observations ascertained that *GDF3* acts in both principal aspects of the stemness state including the promotion of the undifferentiated state in human ESCs, and sustaining the ability of entire differentiation in mouse ESCs [9]. In fact, overexpression of *GDF3* protects the undifferentiated state of ESCs under advanced differentiation conditions [12]. Using the NCCIT cell line, it was revealed that *GDF3* induced expression of the genes pertinent to cell differentiation, which can act as strong tumor suppressors, such as Hox-family genes [11].

**Figure 3.**
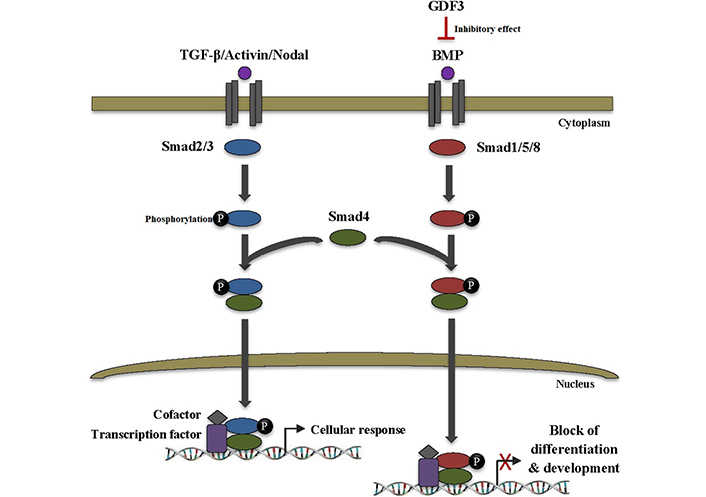
GDF3 inhibits differentiation and development in ESCs. TGF-β superfamily ligands transduce signals via binding to heteromeric complexes of serine/threonine kinase receptors which subsequently recruit and phosphorylate intracellular Smad proteins. Smad2/3 is activated by TGF-β, activin, and nodal ligands, whereas Smad1/5/8 is activated by BMP ligands. Activated Smads then form a heteromeric complex with Smad4. The complex translocates to the nucleus and binds to the specific DNA sequences to regulate the transcription of various target genes through interaction with transcription factors and cofactors, thus leading to cellular response. On the other hand, BMP binding to the receptor can be inhibited by its binding to different extracellular inhibitor proteins, such as GDF3 which ultimately leads to a blocking of the BMP signaling pathway as well as inhibition of vital cell fate decisions, especially differentiation, and development in ESCs. Smad2/3: small mothers against decapentaplegic 2/3

GDF3, which acts as a BMP antagonist and nodal-like agonist, has an atypical structure in the TGF-β superfamily. It is lacking the fourth cysteine of the canonical seven cysteines existent in other family members. This cysteine is importantly implicated in intermolecular interactions between TGF-β ligands, e.g., BMP-4. GDF3 is able to bind to BMP-4 and inactivate it in the extracellular space, thereby blocking of BMP signal transduction pathway. This can lead to the inhibition of differentiation and development as well as other critical cell fate decisions in ESCs (Figure 3) [9, 10]. Moreover, *GDF3* stimulation and knockdown trials manifested the involvement of *GDF3* in the BMP signaling pathway. *GDF3* treatment increased expression of BMP receptor type 2 (BMPR2), while its knockdown downregulated *BMP-2*, *4*, and *7*. Since active BMP signaling in tumors has been proven to be useful for cancer consequences, the GDF3-BMP connection might be investigated in designing new anti-cancer therapeutic modalities [11].

GDF3 was scrutinized to participate in both classical pathways triggered by TGF-β family components. First, it is included in the inhibition of the BMP signaling cascade through direct interaction with extracellular BMP proteins. And second, it is involved in the robust induction of Smad2/3 phosphorylation via binding to activin receptors type IB (ACVRIB), ACVRIC, ACVRIIA, and ACVRIIB in collaboration with necessary co-receptor teratocarcinoma-derived growth factor 1 (TDGF1) [11]. In ESCs that have either activated TGF-β/activin/nodal signaling or lack/low BMP signaling pathways, GDF3 can function to retain both pathways [9].

*GDF3*, as a mammalian-specific TGF-β ligand, is now considered to be a classic *ESC* gene and an indispensable regulator of mammalian embryogenesis [10, 12]. Interestingly, in human ESCs, *GDF3* supports the maintenance of the famous stem cell markers, *OCT4*, *NANOG*, and sex-determining region of Y-chromosome-box transcription factor 2 (*SOX2*) [13]. It has been reported that *GDF3* and *NANOG* are vital for ESC self-renewal and contribute to the early remodeling of the developing embryo [27]. Additionally, *GDF3* is a direct transcriptional target of *NANOG* in embryonic carcinoma cells [28]. High expression of *PYGO2*, the pivotal transcription factor of wingless-type mouse mammary tumor virus integration site (WNT) signaling pathway, was detected in ESCC in correlation with tumor invasion and advanced stages of the disease [29]. On the other hand, concomitant overexpression of spalt like transcription factor 4 (*SALL4*) and *SOX2*, the two main stem cell markers, was elucidated in ESCC in association with tumor invasion and aggressiveness of the disease suggesting coordination of *SALL4* and *SOX2* through WNT/β-catenin pathway in ESCC [30]. Based on this evidence, there are connections between *GDF3* and such master transcriptional factors in the maintenance of the self-renewal and stemness state of ESCs and CSCs. Since the findings of this research illustrated the overexpression of *GDF3* as well as its significant clinicopathological relevance in ESCC, the existence of a similar regulatory network in ESCC and its significant involvement in the progression and invasiveness of the disease may be considered through probable molecular interactions between *GDF3* and other stem cell markers such as *SALL4* and *SOX2* in WNT pathway.

In conclusion, the clinical relevance of *GDF3* expression was presented at the mRNA level in ESCC. To the best of our knowledge, this is the first report revealing the expressional pattern of *GDF3* in ESCC and its significant association with the depth of tumor invasion. These findings may show the impact of CSC marker *GDF3* on tumor progression and aggressiveness of ESCC and identify the potential therapeutic target to inhibit tumor cell invasion. However, the functional role and mechanisms of *GDF3* in ESCC development and progression are still unknown and require further investigation.

## Abbreviations

ACVRIB: activin receptors type IB
BMPs: bone morphogenetic proteins
cDNA: complementary DNA
CSCs: cancer stem cells
ESCs: embryonic stem cells
ESCC: esophageal squamous cell carcinoma
*GAPDH*: glyceraldehydes 3-phosphate dehydrogenase
GDF3: growth and differentiation factor 3
mRNA: messenger RNA
NANOG: Nanog homeobox
PCR: polymerase chain reaction
SALL4: spalt like transcription factor 4
SD: standard deviation
Smad2/3: small mothers against decapentaplegic 2/3
SOX2: sex-determining region of Y-chromosome-box transcription factor 2
T3: tumor invasion level 3
TGF-β: transforming growth factor-beta
WNT: wingless-type mouse mammary tumor virus integration site

## Declarations

### Copyright

© The Author(s) 2023.
